# Snow-mediated plasticity does not prevent camouflage mismatch

**DOI:** 10.1007/s00442-020-04680-2

**Published:** 2020-06-24

**Authors:** Alexander V. Kumar, Marketa Zimova, James R. Sparks, L. Scott Mills

**Affiliations:** 1 Wildlife Biology Program, University of Montana, Missoula, MT 59812 USA; 2 Department of Forestry and Environmental Resources, Program in Fisheries, Wildlife and Conservation Biology, North Carolina State University, Raleigh, NC 27695-7617 USA; 3 School for Environment and Sustainability, University of Michigan, Ann Arbor, MI 49109 USA; 4 Missoula Field Office, Bureau of Land Management, Missoula, MT 59804 USA; 5 Wildlife Biology Program and Office of the Vice President for Research and Creative Scholarship, University of Montana, Missoula, MT 59812 USA

**Keywords:** Adaptive rescue, Phenotypic plasticity, Behavioral plasticity, Climate change, Molt phenology

## Abstract

Global reduction in snow cover duration is one of the most consistent and widespread climate change outcomes. Declining snow duration has severe negative consequences for diverse taxa including seasonally color molting species, which rely on snow for camouflage. However, phenotypic plasticity may facilitate adaptation to reduced snow duration. Plastic responses could occur in the color molt phenology or through behavior that minimizes coat color mismatch or its consequences. We quantified molt phenology of 200 wild snowshoe hares (*Lepus americanus*), and measured microhabitat choice and local snow cover. Similar to other studies, we found that hares did not show behavioral plasticity to minimize coat color mismatch via background matching; instead they preferred colder, snow free areas regardless of their coat color. Furthermore, hares did not behaviorally mitigate the negative consequences of mismatch by choosing resting sites with denser vegetation cover when mismatched. Importantly, we demonstrated plasticity in the initiation and the rate of the molt and established the direct effect of snow on molt phenology; greater snow cover was associated with whiter hares and this association was not due to whiter hares preferring snowier areas. However, despite the observed snow-mediated plasticity in molt phenology, camouflage mismatch with white hares on brown snowless ground persisted and was more frequent during early snowmelt. Thus, we find no evidence that phenotypic plasticity in snowshoe hares is sufficient to facilitate adaptive rescue to camouflage mismatch under climate change.

**Electronic supplementary material:**

The online version of this article (10.1007/s00442-020-04680-2) contains supplementary material, which is available to authorized users.

## Introduction

The detrimental effects of climate change on the fitness of wild populations may be ameliorated by adaptation (Hoffmann and Sgró 2011). Local adaptation to rapid environmental change may occur through both evolution via natural selection (i.e., evolutionary rescue (Vander Wal et al. 2013)) and phenotypic plasticity (Ghalambor and Martin 2001). Although both mechanisms are possible and can interact (Forsman 2015), phenotypic plasticity may provide the most rapid adaptive response in the face of climate change (Gienapp et al. 2008; Beever et al. 2017; Snell-Rood et al. 2018). Climate change-induced plasticity in the timing of life history events has been demonstrated in various taxa including amphibians (Parmesan 2007), butterflies (Parmesan 2007), birds (Przybylo et al. 2000), and mammals (Ozgul et al. 2010; Lane et al. 2012). However, the scope for plastic responses to maintain fitness under climate change is largely unknown.

Climate change can affect seasonal phenological processes through shifts in abiotic drivers such as temperature (Both and Visser 2001; Kudo and Ida 2013) and precipitation (both rain (Penuelas et al. 2012; Cohen et al. 2018) and snow (Sheriff et al. 2011; Lane et al. 2012; Rickbeil et al. 2019)). Snow is particularly relevant because a reduction in number of days with snow cover is one of the most consistent and widespread signals of climate change in the northern hemisphere (Pederson et al. 2011; Kunkel et al. 2016; Zhu et al. 2019). Snow declines have a direct impact on organisms adapted to seasonal environments (Williams et al. 2015; Hock et al. 2019; Shipley et al. 2019), including a diverse group of birds and mammals that molt from summer brown to winter white annually to increase crypsis against snow (Mills et al. 2018; Zimova et al. 2018). For these species, reduced snow duration can increase camouflage mismatch, a striking and direct climate change stressor (Mills et al. 2013, 2018), which results in white animals suffering higher predation rates against a dark snowless background (Zimova et al. 2016; Atmeh et al. 2018; Wilson et al. 2018).

The snowshoe hare (*Lepus americanus*), an important prey species for a plethora of carnivores including Canada lynx (*Lynx canadensis)*, undergoes coat color molts across most of its range (Nagorsen 1983; Jones et al. 2018; Mills et al. 2018). Snowshoe hares experience 7–12% reductions in weekly survival when their coat color is mismatched against the background color (Zimova et al. 2016; Wilson et al. 2018). These fitness costs of camouflage mismatch are sufficient to cause severe population declines in absence of future adaptive responses (Zimova et al. 2016). In fact, hares have already experienced range contractions linked to reduced snow duration and mismatch-related mortality (Burt et al. 2017; Diefenbach et al. 2016; Sultaire et al. 2016).

Plasticity in both molt phenology and in behaviors may reduce or eliminate the fitness costs to mismatched hares. The initiation of the seasonal color molts is largely driven by photoperiod (Lyman 1943) with potential plasticity in the population molt rate modulated by temperature and snow (Zimova et al. 2018). The modulating effect of temperature on seasonal color change in several species has been repeatedly demonstrated in laboratory (Rothschild 1942; Rust 1962) and field settings (Watson 1963; Flux 1970; Zimova et al. 2014, 2020a; Kumar 2015), although its physiological mechanism or fitness consequences are unknown. Whether or how snow can modify molt phenology is much less clear (Grange 1932; Jackes and Watson 1975; Nagorsen 1983; Zimova et al. 2014).

Snow may affect molt phenology indirectly via temperature or directly via light-mediated hormone shifts. Snow may influence temperature by creating a warmer, more thermally stable subnivium space (Goodrich 1982; Pauli et al. 2013) or by its high albedo lowering temperatures (Namias 1985; Choi et al. 2010). The direct physiological effects of snow on molt phenology may occur if light reflected off snow mediates melatonin levels through the same neuroendocrine pathway that regulates photoperiodic systems (Goldman 2001; Schwartz et al. 2001). Although snow has not been definitively linked to molt phenology, it is known to influence other phenological processes such as hibernation (Sheriff et al. 2011; Lane et al. 2012), reproduction (Liebezeit et al. 2014) and migration (Rickbeil et al. 2019).

In addition to plasticity in phenology, animals may directly and rapidly adapt to climate change through other types of behavioral plasticity (Beever et al. 2017; Zimova et al. 2018; Stevens and Ruxton 2019). For seasonally color molting species, plasticity in behaviors may reduce camouflage mismatch per se, or reduce the fitness consequences of mismatch. In a classic example of behavioral plasticity to minimize mismatch, molting ptarmigan (*Lagopus lagopus*) preferentially forage in areas that maximize their crypsis during their color molts (Steen et al. 1992). Other plastic responses might include behaviors such as resting in dense vegetation, where predator detection and capture rates are lower (Ivan and Shenk 2016). Snowshoe hares are capable of exhibiting behaviors to assess and reduce predation risk; for example, under a full moon, hares increase their use of high cover areas (Gilbert and Boutin 1991; Gigliotti and Diefenbach 2018) or decrease their movement (Griffin et al. 2005). Although a general preference for dense cover is a hallmark of snowshoe hare biology (Adams 1959; Wolff 1980; Litvaitis et al. 1985), the two existing studies that examined concealment in relation to mismatch provide contrasting results (Litvaitis 1991; Zimova et al. 2014).

The central aim of this study is to determine the ability and scope for phenotypic plasticity to facilitate adaptive responses to camouflage mismatch. First, we quantified plasticity in color molt phenology of snowshoe hares and the extent to which it is driven by snow cover. We predicted that hares molt to white earlier and faster during snowier falls and become brown later and slower during snowier springs. Second, we tested whether hares exhibit behavioral plasticity through either choice of background to minimize mismatch or choice of habitat structure to increase concealment. Finally, we evaluated whether these possible avenues of phenotypic plasticity are sufficient to reduce the occurrence of camouflage mismatch.

## Methods

### Study areas

Fieldwork for this study was conducted in two areas of the Upper Blackfoot region of western Montana, USA on land managed by the Bureau of Land Management; see (Kumar et al. 2018) for details. Marcum Mountain (Lat. = 46.99°, Long. = − 112.91°) and Chamberlain Creek (Lat. = 46.96°, Long. = − 113.24°) are approximately 30 km apart at similar elevations (approximately 1450–1700 m.a.s.l.). Both areas have dominant tree species of western larch (*Larix occidentalis*) (approximately 25–50% of total stems) interspersed with Douglas fir (*Pseudotsuga menziesii*), subalpine fir (*Abies lasiocarpa*), lodgepole pine (*Pinus contorta*) and Engelmann spruce (*Picea engelmannii*) and an herbaceous understory. For the analysis on the influence of snow on molt phenology only, we also used data previously collected at two additional study sites: (1) Seeley was approximately 40 km away with similar vegetation types and elevation (1400 m.a.s.l.) (Mills et al. 2013; Zimova et al. 2014), (2) Gardiner was approximately 300 km away at about twice the elevation (2400–2700 m.a.s.l.) with cooler temperatures and longer durations of snow cover (Zimova et al. 2014). Likely predators of snowshoe hares at all sites include Canada lynx, bobcat (*Lynx rufus*), cougar (*Puma concolor*), coyote (*Canis latrans*), red fox (*Vulpes vulpes*), American marten (*Martes americana*), long-tailed weasel (*Mustela frenata*), golden eagle (*Aquila chryseatos*), great horned owl (*Bubo virginianus*), barred owl (*Strix varia*), northern goshawk (*Accipiter gentilis*), and red-tailed hawk (*Buteo jamaicensis*).

### Capture/handling

We live-trapped snowshoe hares during fall 2012 and summer and winter 2013–2014 using live traps (51 × 18 × 18 cm, Tomahawk Live Trap Company, Tomahawk, WI) baited with alfalfa cubes and apples. We marked all hares > 500 g with a unique numbered ear tag and a VHF radio-collar (Wildlife Materials, Murphysboro, IL) weighing < 40 g. All capture and handling procedures were approved by the University of Montana Animal Care and Use Committee (permit no. AUP 021-13SMECS-050613).

### Snowshoe hare molt phenology and resting site selection

Using VHF radio-telemetry, we tracked 49 individual snowshoe hares and obtained 280 locations during the fall brown-to-white and the spring white-to-brown molts. We used the VHF signal to visually detect hares during the day (typically between 10:00 and 16:00) usually at a distance of 3–10 m. Observations typically occurred at resting sites, “forms”, (94% of all observations), which usually overlap the habitat used by foraging hares (Ferron and Ouellet 1992; Hodges 2000). We determined if the hare was resting in a form (as opposed to merely transitioning through) by examining their location for an indentation in the vegetation or snow and fresh feces. We followed a standardized protocol of visual observation and photography to estimate hare percent white: (0%, 5%, 20%, 40%, 60%, 80%, 95%, and 100%) (Mills et al. 2013). Hare percent white was typically estimated by two observers in the field and, in the cases of uncertainty, a single observer (AVK) used photographs to make the final determination. We considered a hare to be molting when it was > 0% but < 100% white.

Each time we located a hare in a form, we recorded associated resting site characteristics. We estimated snow cover at each hare location in 20% increments as the percent of the ground covered with snow. We measured snow cover at both 1- and 10-m plot radii centered on the hare form to capture different scales at which crypsis may be perceived (Zimova et al. 2014). We computed color contrast (hare percent white - snow cover), and defined mismatch as occurring when the absolute value of color contrast was ≥ 60% (Mills et al. 2013). We further differentiated mismatch between white hares found on brown snowless backgrounds (white hare mismatch) and brown hares found on snowy backgrounds (brown hare mismatch). We recorded the temperature at the hare form with a handheld weather meter (Kestrel 2000, Nielsen-Kellerman, Boothwyn, PA). We used a circular plot of 5-m radius centered on the hare form to quantify the total number of stems (> 1 m tall and > 2.5 cm diameter at breast height) of all trees and shrubs (Lewis et al. 2011; Kumar et al. 2018).

We used a paired, used–available design to (1) test for the ability of hares to minimize mismatch or increase concealment and (2) unravel a causal (snow influencing the molt) relationship versus a correlative one (white hares preferring snow). We paired each hare location (used) with an available location in a random direction and distance (weighted by hare home range size, see Appendix S1) from each hare location. We recorded snow cover, temperature and stem counts at each available point immediately after sampling the paired used point.

### Statistical analyses

We used mixed-effect models using package “lme4” (Bates et al. 2015) in Program R (version 2.15.1, R Development Core Team 2012) to evaluate snowshoe hare molt phenology association with snow and hare resting site selection. We standardized all predictors to a mean of 0 and a standard deviation (SD) of 1 to facilitate comparisons between models; when predictors were strongly correlated (> 0.7), we included only the predictor that best fit the data using a linear model (Dormann et al. 2013). Because all the predictors were biologically informed, we only ran the full models (Bolker et al. 2009), which included the response and all uncorrelated fixed and random effects. For comparison purposes, and to more explicitly consider model fit, we also model averaged by AICc using the full model and all combinations of the reduced models (Appendix S2) and found no major differences in predictor estimates. To help interpret effect sizes, we present effects in terms of how a one SD change in the predictor would affect the response, in addition to providing the predictor means and standard errors.

#### Snowshoe hare molt phenology

We quantified hare molt phenology during spring 2013, 2014 and fall 2013 at Marcum and spring 2014 and fall 2013 at Chamberlain (insufficient data prevented estimation of molt initiation and completion dates for some year/site combinations). To accommodate incomplete detection and high hare mortality, we used a Bayesian mixed-effect change point analysis to estimate the mean population molt initiation and completion dates (Mills et al. 2013; Zimova et al. 2014). We used a repeated-measures model with individual hare as the random effect to avoid the pseudoreplication that would occur by considering the repeated measures of the same individual as independent samples. The model was fit with Markov Chain Monte Carlo (MCMC) in OpenBugs with uninformative priors. All models were run with five chains of 100,000 iterations (discarding 10,000 burn-ins), with the Gelman–Rubin statistic indicating chain convergence ($\widehat{R}$ ≤ 1.1). We calculated initiation and completion dates of the molt and the resultant 95% credible intervals for each site and season separately. We also estimated area and year specific snow cover by averaging the snow cover estimates taken multiple times a week at used and available sites. We extrapolated estimates for missing days using a moving average yielding daily snow cover estimates (Mills et al. 2013; Zimova et al. 2014).

#### Snow influence on molt phenology

We used a mixed-effect modeling approach to evaluate whether snow cover was positively associated with hare percent white during the molt. We included only observations when hares were molting (i.e., 5–95% white). In addition, for this question only, we were able to add data collected from 2009 to 2012 using the same protocols from other sites in Montana (Mills et al. 2013; Zimova et al. 2014), increasing our sample size to 200 individual hares and 1313 hare locations. We included a fixed effect for date to prevent attributing to snow cover any changes in the hare percent white that were actually due to season (i.e., the occurrence of both white hares and snow is expected to increase over time in the fall). Thus, all seasonal changes were absorbed by the date fixed effect, leaving only daily variation in hare percent white to be explained by the model; though rigorous, this approach will produce smaller effect sizes. Because the fixed effect of date on hare percent white would have a positive slope in the fall and negative slope in the spring, we analyzed spring and fall separately. The full model consisted of hare percent white as the response, fixed effects for date and the standardized measurement of snow cover at 10 m, and random effects for individual hare, year and region.

#### Hare resting site selection

We tested for hare resting site selection by fitting a generalized linear model with a binomial distribution to examine the differences between forms used by hares and the paired available locations. The full model consisted of used versus available locations as the response and the standardized measurements of number of stems, temperature at the form, snow at 1 m and all of their one-way interactions as fixed effects and a random effect for individual hare. We expressed the results as odds ratios on the real scale. We also tested for differences in resting site selection by subsetting the data for instances when hares were mismatched (color contrast ≥ 60%) compared to when they matched their background (color contrast < 60%).

## Results

### Snowshoe hare molt phenology and mismatch

**Fig. 1:**
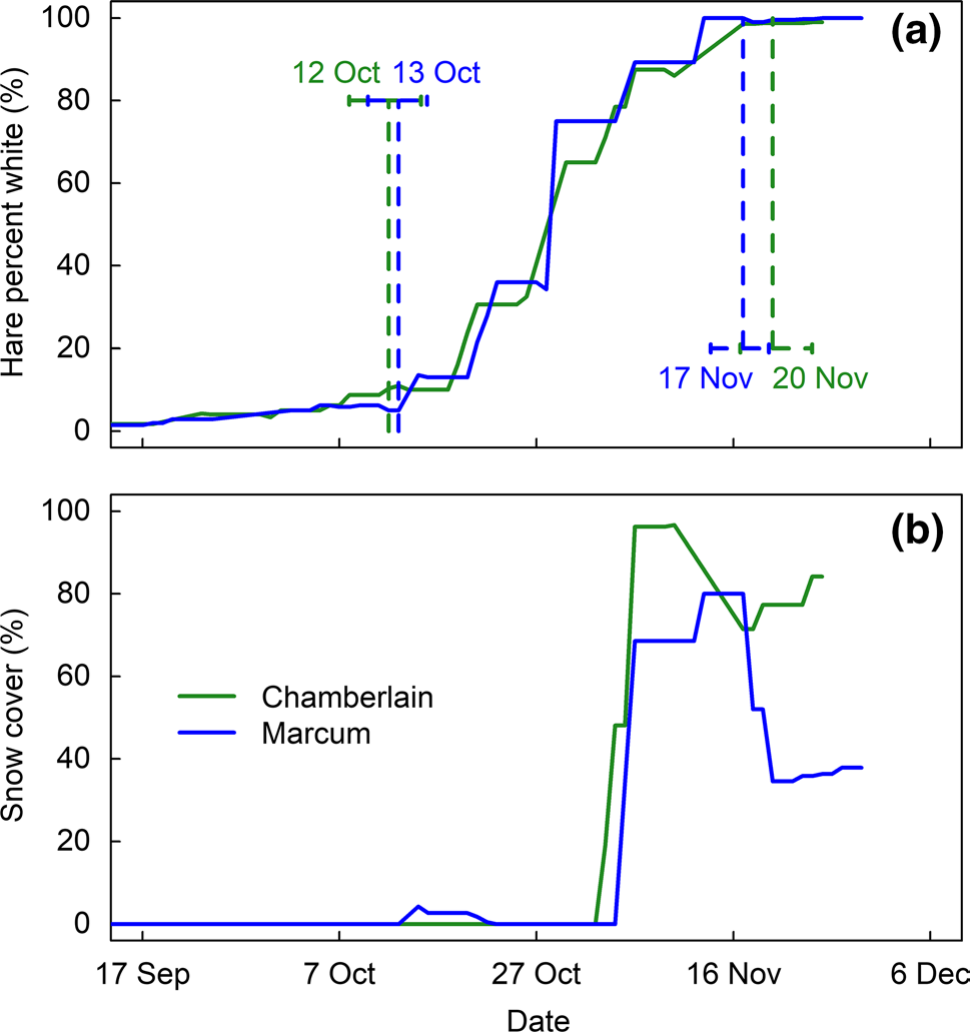
Fall coat color molt phenology and snow cover in 2013 in two study areas in western Montana, north-central USA. Blue line denotes Marcum study area and green line denotes Chamberlain study area. **a** Mean weekly coat color of 38 snowshoe hares at two different study areas. Dotted lines indicate mean molt initiation and completion dates and the 95% credible intervals. **b** Area-specific averaged weekly snow cover based on a moving average of all 10-m-radius snow cover estimates for each hare per observation

**Fig. 2:**
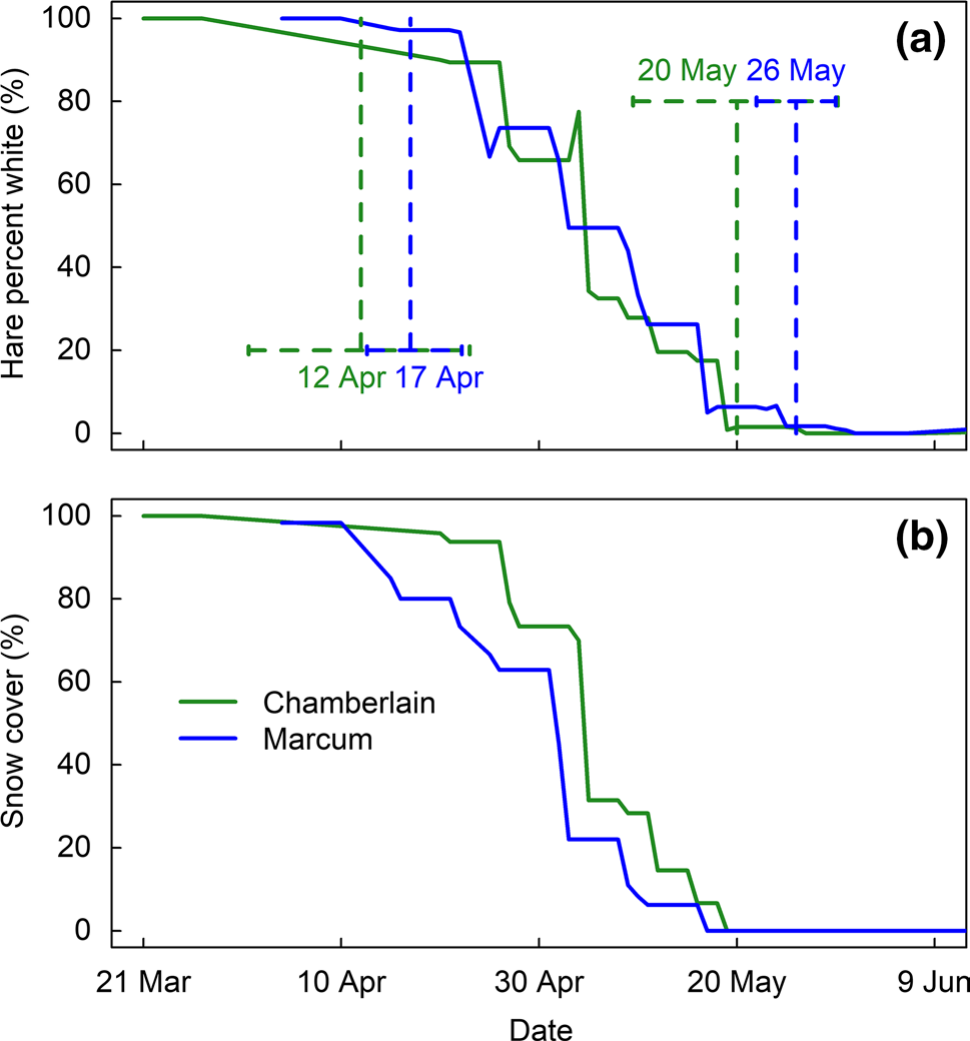
Spring coat color molt phenology and snow cover in 2014 in two study areas in western Montana, north-central USA. Blue line denotes Marcum study area and green line denotes Chamberlain study area. **a** Mean weekly coat color of 16 snowshoe hares at two different study areas. Dotted lines indicate mean molt initiation and completion dates and the 95% credible intervals. **b** Area-specific averaged weekly snow cover based on a moving average of all 10-m-radius snow cover estimates for each hare per observation

We found no significant differences (i.e., 95% credible intervals overlap) in either the estimated initiation or completion dates of snowshoe hare molts between the two study areas during the same season (Figs. 1a, 2a). Consequently, the hare molt duration for both study areas in fall 2013 and spring 2014 was similar: Marcum (36, 40 days) and Chamberlain (40, 39 days). Phenology of snow cover was also similar in both areas in fall (Fig. 1b) and spring (Fig. 2b).

**Fig. 3:**
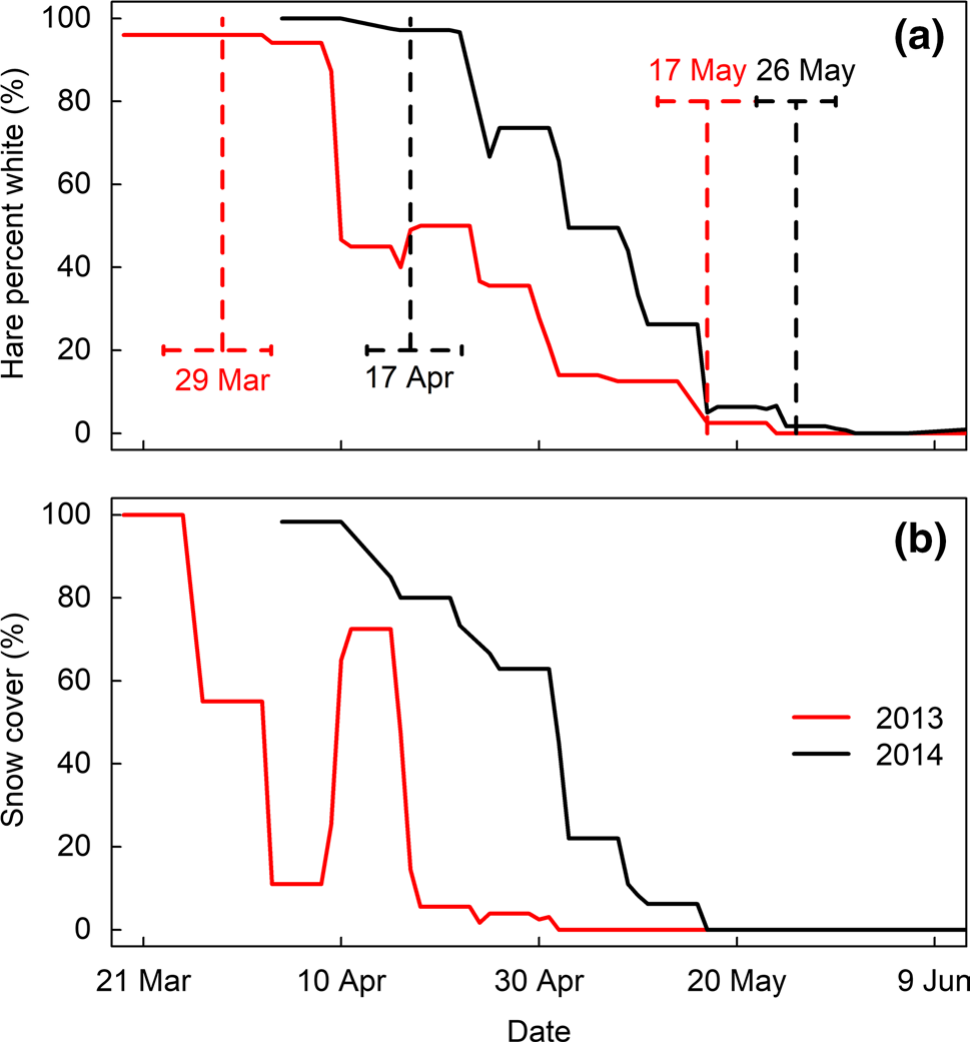
Shifts in spring coat color molt phenology follow spring snow cover at the Marcum study site. Red line denotes 2013 and black line denotes 2014. **a** Mean weekly coat color of 23 snowshoe hares. Dotted lines indicate mean molt initiation and completion dates and the 95% credible intervals. **b** Area-specific averaged weekly snow cover based on a moving average of all 10-m-radius snow cover estimates for each hare per observation

At Marcum, where we monitored molts over two springs, we found significant differences in spring molt phenology between the two years, with each spring molt phenology tracking snow cover phenology (Fig. 3). In 2013, hares initiated the molt 20 days earlier than in 2014 and completed 10 days earlier (Fig. 3a). The more rapid molt to brown in 2013 corresponded with a shorter spring snow duration (snow melted earlier and faster in 2013) (Fig. 3b).

**Fig. 4:**
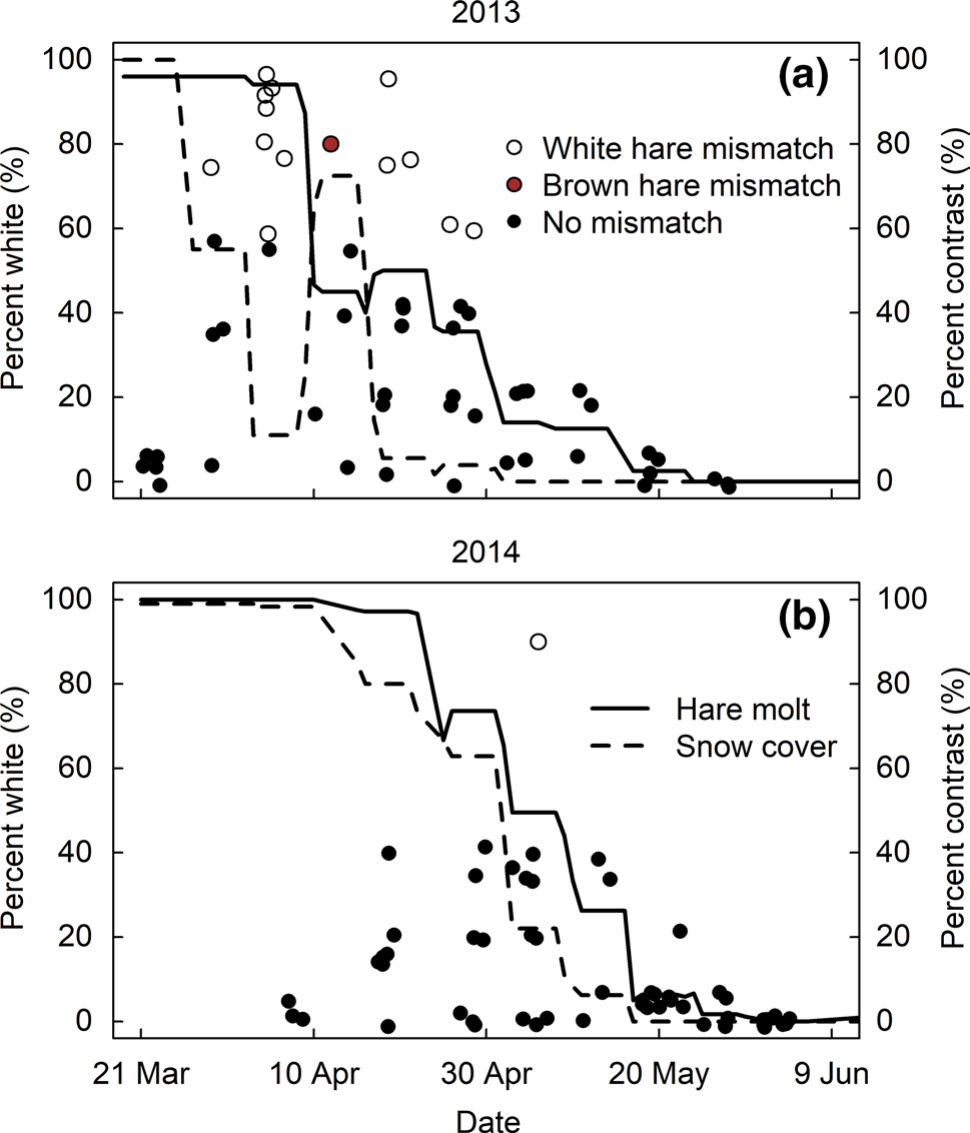
Early snowmelt results in increased camouflage mismatch in hares in western Montana, north-central USA. **a** Color contrast and resulting occurrence of mismatch from 56 observations of 15 individuals in 2013 at Marcum study area. Percent white on the y-axis represents both hare percent white and percent snow cover at 10-m-radius. White points indicate white hares mismatched on brown backgrounds, brown points indicate brown hares mismatched on white backgrounds and black points indicate no mismatch. Percent contrast = hare percent white - percent snow cover. Mismatch occurs when contrast ≥ 60%. **b** Color contrast and mismatch resulting from 57 observations of 8 individuals in 2014 at Marcum study area

Despite the plasticity in molt phenology across years, the earlier snowmelt year of 2013 nevertheless resulted in increased camouflage mismatch. In 2013, we detected 13 occurrences of white hare mismatch (whites hares on snowless backgrounds) and one occurrence of brown hare mismatch (brown hares on snow), based on 56 observations of 15 individuals (Fig. 4a). The 13 occurrences of white hare mismatch were observed in 11 different individuals. By contrast, in the later snowmelt year of 2014, we detected one occurrence of white hare mismatch, based on 57 observations of 8 individuals (Fig. 4b).

### Snow influence on molt phenology

We found a strong effect of snow cover on snowshoe hare molt phenology using data from 200 hares over 6 years, even after controlling for date. An increase of one SD of percent snow cover (36% in fall and 44% in spring) was associated with an increase in hare percent white by 3% (SE = 0.8%) in fall and 11% (SE = 1.3%) in spring.

### Hare resting site selection

Overall, molting hares had strong preference for resting sites with more total stems, less snow cover and colder temperatures (Table 1). An increase in the number of stems by one SD (23 stems) increased the odds of hare use by 1.5 times (95% CI 1.3–1.8). A decrease in percent snow cover by one SD (35%) increased the odds of hare use by 1.4 times (95% CI 1.0–1.8). A decrease in temperature by one SD (8.0 °C) increased the odds of hare use by 1.3 times (95% CI 1.0–1.5). Whether or not an individual was mismatched did not affect these microhabitat preferences, suggesting a lack of behavioral plasticity to reduce mismatch or its fitness costs. Because hares consistently preferred snow-free (brown) resting sites, we found no evidence that white hares background match via preferentially choosing snowy areas to maintain crypsis. In fact, white hares tend to select resting sites with more bare ground even though brown ground leads to mismatch. Likewise, similar overall preference for resting sites with denser cover (more stems)—regardless of whether or not a hare was mismatched—indicated that mismatched hares did not choose microhabitats that potentially provided more protection from predation.

**Table 1:** Snowshoe hare resting site selection in western Montana, north-central USA

Predictor	Used (SE)	Available (SE)
Number of stems	35.86 (1.23)	26.87 (1.24)
Temperature (°C)	9.04 (0.42)	9.51 (0.45)
Percent snow cover	12.51 (1.58)	22.36 (2.09)

Used sites correspond to locations of hares found in their forms. Each available site corresponds to a random location (Appendix S1). At each site, a 5-m-radius plot was established and total number of stems (> 1 m tall and > 2.5 cm diameter at breast height), temperature and percent snow cover were measured. Mean values of each predictor are given with standard error denoted by parentheses

## Discussion

Although phenotypic plasticity can enable rapid adaptation to climate change (Gienapp et al. 2008; Beever et al. 2017; Snell-Rood et al. 2018), ecologists often lack an understanding of mechanisms underlying adaptive phenotypic plasticity, and the extent to which plasticity can reduce or eliminate phenological mismatches between seasonal biotic processes and abiotic climate variables. Focusing on snowshoe hares, a snow-adapted mammal, we provide strong evidence that snow cover drives the observed phenotypic plasticity in molt phenology. However, we also find that this snow-mediated plasticity is insufficient to substantially reduce mismatch between white hares and snowless backgrounds. Furthermore, we find no evidence that snowshoe hares perceive coat color mismatch and adjust their behavior to reduce mismatch or its consequences.

At the Marcum site, where we could follow the spring molt across two years of very different snow duration, we documented phenological shifts in both the initiation and rate of the snowshoe hare spring molt in the direction of annual changes in snow cover (Fig. 3). The much shorter snow duration of 2013 at the Marcum site was corroborated by a nearby weather station (approximately 90 km away in Missoula) which recorded roughly 25% lower snow accumulation compared to 2014 (123 cm in 2013, 167 cm in 2014). In that early snow melt year of 2013, hares began their spring molt almost 3 weeks earlier and became brown about 10 days earlier than in 2014. Accordingly, the 2013 molt took about 50 days, 10 days longer than in 2014 and in other studies (Zimova et al. 2014, 2020a).

In addition to documenting phenological shifts tracking snow cover (Fig. 3), we directly modeled the effect of local snow cover on hare percent white using data spanning 6 years at four study areas separated by up to 300 km, confirming snow as a driver of the observed phenological shifts. The snow effects on molt phenology were evident even after controlling for date, ensuring any changes in the hare percent white were attributable to snow cover and not actually due to season. Importantly, the positive association between snow and hare percent white did not arise from a behavioral preference for snow. On the contrary, snowshoe hares preferred areas with more bare ground, consistent with previous field-based findings at other study sites (Zimova et al. 2014). Thus, associations between snow and hare percent white arise from snow directly affecting hare molt phenology as opposed to behavioral plasticity of white hares preferring snowy areas. Therefore, our findings imply that while photoperiod is the core modulator of the seasonal color molt, snow presence per se may facilitate plasticity in spring molt phenology, perhaps via the same neural pathways that perceive changes in photoperiod (Goldman 2001; Schwartz et al. 2001).

Although we established snow-driven plasticity in hare molt phenology, we found no evidence of behavioral plasticity to choose resting areas that reduce coat color mismatch or the associated increased predation risk. We found that regardless of coat color or mismatch, hares preferred areas with no snow and resting sites with dense stems. Thus, hares do not preferentially choose to background match when snow is patchy, or reduce the negative survival costs of mismatch (Zimova et al. 2016; Wilson et al. 2018) through increased concealment. The lack of plasticity to increase concealment when mismatched is consistent with a field study using similar methods to ours with radio-collared hares (Zimova et al. 2014). By contrast, another study that examined hare distributions using baited live traps found that mismatched hares were more often trapped in areas with denser cover (Litvaitis 1991). Hare overall preference for areas with more stems is likely due to the predation protection it affords (Ivan and Shenk 2016); however, the reason hares prefer non-snowy areas is unknown. Possible explanations for hare preference for bare ground include locomotive efficiency and thermoregulatory constraints.

Consistent with thermoregulation influencing hare site selection, we found support for hare preference of colder microhabitats. Because thermal properties such as increased insulation accompany the winter white molt (Walsberg 1991; Sheriff et al. 2009), molting hares may prefer colder areas when ambient temperatures are warm. In addition to its effect on hare resting site selection, temperature may also have a weak influence on hare molt phenology (Watson 1963; Nagorsen 1983; Zimova et al. 2014, 2020a; Kumar 2015). Field-based tests of the direct effects of temperature on coat color molt are not yet possible due to the absence of a physiologically based connection between the trait and a relevant temperature metric (e.g., daily mean temperature, monthly maximum temperature, number of days above freezing, etc.) or appropriate temporal window.

Might the plasticity in both the initiation and rate of the molt provide a means for adaptive rescue if hare populations begin to decline due to increased mismatch resulting from shortening snow seasons? The observed difference in spring snow duration between 2013 and 2014 was approximately 20 days. This is less than the 29–35-day reduction in snow duration predicted in this area by mid-century, under either the medium–low or high greenhouse gas concentration scenarios, respectively (Mills et al. 2013). Although the mean population molt phenology (initiation and rate of molt) shifted in the direction of the rapid 2013 snowmelt, we nevertheless detected white hare mismatch in 23% of our observations (Fig. 4a), contrasted with just 2% of observations in 2014 (Fig. 4b). Moreover, analysis of raw data from a previously published study (Mills et al. 2013) revealed white hare mismatch in 15% of observations from an early snowmelt year (2010) compared to just 1% of the observations in the later snowmelt year (2011). In short, we find no evidence that snow-driven plasticity in molt phenology will prevent increased mismatch under reduced snow duration. Therefore, phenotypic plasticity in hare molt phenology is unlikely to facilitate adaptive rescue, a finding consistent with other studies that find phenotypic plasticity insufficient to enable long-term species persistence under climate change (e.g., Davis et al. 2017; Schmaljohann and Both 2017; Radchuk et al. 2019).

The extent to which phenotypic plasticity in molt phenology is driven by snow in the other 20 color molting species is unknown. However, the effects of extrinsic factors on molt phenology are similar in other mammalian color molting species (Zimova et al. 2018). Therefore, it is possible that snow plays a mediating role across taxa, although sympatric interspecific differences in molt phenology do occur (Davis et al. 2019). In addition to color molting species, snow has also been shown to affect other phenological processes across diverse taxa. For example, in both Columbian and Arctic ground squirrels, hibernation emergence is influenced by snow cover (Sheriff et al. 2011; Lane et al. 2012). Likewise, snow melt drives the onset of vegetative growth, (Inouye et al. 2000) and the seasonal migration patterns of elk (Rickbeil et al. 2019). Finally, snow melt was the most important predictor explaining clutch initiation advancement for arctic shorebird and passerine species, explaining more variation in phenology than green-up (Liebezeit et al. 2014). Thus, snow-mediated plasticity in phenological processes appears to be more common than previously expected.

Although the scope of our inference strictly applies to only snowshoe hares in western Montana, it builds on a substantial body of work establishing this species as a powerful model system to reveal general principles for how species might adapt to climate change (e.g., Jones et al. 2018, 2020a, b, Mills et al. 2013, 2018; Sultaire et al. 2016; Wilson et al. 2018; Zimova et al. 2020a, b). While we find for hares that persistence under rapid environmental change will likely depend less on phenotypic plasticity and more on the potential for evolutionary change, an urgent priority is to determine across all species the relative importance of these modes of adaptive rescue (Bay et al. 2017; Diamond et al. 2017; Manhard et al. 2017; Mills et al. 2018).

## Electronic supplementary material

Below is the link to the electronic supplementary material.

Supplementary material 1 (DOCX 13 kb)

Supplementary material 2 (DOCX 21 kb)

## Data Availability

The data reported in the paper are archived in the Dryad Digital Repository at 10.5061/dryad.7d7wm37s8.
