# Supplementary material for: Snow-mediated plasticity does not prevent camouflage mismatch
Source: Oecologia. 2020 Jun 24;194(3):301–10. doi: 10.1007/s00442-020-04680-2 (PMC7644448; doi:10.1007/s00442-020-04680-2)
Supplement: Supplementary file 1 — Supplementary material 1 (DOCX 13 kb) [file 442_2020_4680_MOESM1_ESM.docx]

**Appendix S1.** Estimating home range and determining available but unused locations.

We analyzed telemetry locations for snowshoe hares in western Montana from 1999-2002 (P. Griffin, unpublished data). We then used these locations to calculate 95% kernel utilization distribution home ranges for hares in Program R using Package “adehabitat”. We separately estimated these home ranges for spring (March-May) and fall (September-December). These months correspond to the times during which hares undergo coat color molts. Next we calculated the mean kernel area and subsequent mean radius assuming a circular home range. We tested the validity of this assumption by plotting the individual home ranges and visually examining them. Most were circular. The mean radius was 176m for spring and 187m for fall. We decided that these differences between spring and fall were not biologically significant so we collapsed them into one mean radius of about 180m for both seasons.

Next we used a half normal distribution to generate values that had 95% of the values less than 180. We used the values generated by the half normal distribution to determine the distance between the hare’s location and the location of its available but unused point. By weighting values based on the half normal distribution we skewed the available points closer to where the hare was located and left no upper bound. Essentially the available point could be any distance from the known point with decreasing probability further from the known point. Values less than 10 meters away from the known point were excluded to prevent overlap with the 5 m circular radii plots. We used a random compass bearing to determine the direction from the known point to the available point.
