# Supplementary material for: Snow-mediated plasticity does not prevent camouflage mismatch
Source: Oecologia. 2020 Jun 24;194(3):301–10. doi: 10.1007/s00442-020-04680-2 (PMC7644448; doi:10.1007/s00442-020-04680-2)
Supplement: Supplementary file 2 — Supplementary material 2 (DOCX 21 kb) [file 442_2020_4680_MOESM2_ESM.docx]

**Appendix S2.** Model averaged results.

For our main analyses, we only ran the full models, which included the response and all plausible fixed and random effects. We decided against including model averaging in our main results because all of our predictors were biologically informed and the interpretation of the parameters changes when the model changes. However, as model averaging is still an accepted technique and more explicitly considers model fit, we also decided to model average the results. In addition to the full model, we ran a set of models including all possible combinations of the fixed effects weighted by Akaike information criterion corrected for small sample size (AICc). The random effects were always included to absorb known causes of variation. The model average results were then compared to the results from the full model (Tables 1 & 2). They do not differ in a biologically meaningful way.

Table S1. Comparison of model averaged results with results from only the full model to explore snowshoe hare microsite selection. Model average results were ranked by Akaike’s Information Criterion adjusted for small sample size (AICc). Predictor estimates were averaged over all models in which the predictor occurred. The full model was: use ~ snow + stems + temp + wind + temp*snow + temp*wind + snow*wind + (1|tag). None of the interactions were significant. Standard error indicated in parentheses. Summary statistics of parameters given before standardization.

| Predictor | Min | Mean | Max | Full Model Estimate (SE) | Model Averaged Estimate (SE) | Sample  Size |
| --- | --- | --- | --- | --- | --- | --- |
| snow (%) | 0 | 17.43 | 100 | -35.51% (18.52%) | -45.51% (16.36%) | 341 |
| stems (#)  temp (^o^C) | 0  -13.2 | 31.36  9.28 | 203  28.9 | 47.33% (5.93%)  -25.38% (12.00%) | 48.38% (5.89%)  -26.69% (11.94%) | 341  341 |
| wind (m/s) | 0 | 0.11 | 0.8 | -12.71% (9.76%) | -14.41% (9.76%) | 341 |

Table S2. Comparison of model averaged results with results from only the full model to explore the effect of snow on the snowshoe hare molt. Model average results were ranked by Akaike’s Information Criterion adjusted for small sample size (AICc). Predictor estimates were averaged over all models in which the predictor occurred. The full model was: white ~ snow10 + date + (1|tag) + (1|year) + (1|region). Separate models were run for spring and fall. Standard error indicated in parentheses. Summary statistics of parameters given before standardization.

| Predictor | Min | Mean | Max | Full Model Estimate (SE) | Model Averaged Estimate (SE) | Sample Size |
| --- | --- | --- | --- | --- | --- | --- |
| snow10 (spring) | 0 | 53.63 | 100 | 10.74 (1.27) | 10.74 (1.27) | 644 |
| date (spring) | 74 | 121.2 | 194 | -31.30 (1.48) | -31.30 (1.48) | 644 |
| snow10 (fall) | 0 | 21.11 | 100 | 2.96 (0.82) | 2.96 (0.82) | 669 |
| date (fall) | 258 | 293.8 | 335 | 29.05 (0.79) | 29.06 (0.80) | 669 |
